# Phylogenetic analysis of family *Neisseriaceae* based on genome sequences and description of *Populibacter corticis* gen. nov., sp. nov., a member of the family *Neisseriaceae,* isolated from symptomatic bark of *Populus × euramericana* canker

**DOI:** 10.1371/journal.pone.0174506

**Published:** 2017-04-13

**Authors:** Yong Li, Han Xue, Sheng-qi Sang, Cai-li Lin, Xi-zhuo Wang

**Affiliations:** 1 The Key Laboratory of State Forestry Administration on Forest Protection, Research Institute of Forest Ecology Environment and Protection, Chinese Academy of Forestry, Beijing, China; 2 Agricultural High and New Technology Development Center of Puyang, Puyang, China; Universidade de Coimbra, PORTUGAL

## Abstract

Two Gram-stain negative aerobic bacterial strains were isolated from the bark tissue of *Populus × euramericana*. The novel isolates were investigated using a polyphasic approach including 16S rRNA gene sequencing, genome sequencing, average nucleotide identity (ANI) and both phenotypic and chemotaxonomic assays. The genome core gene sequence and 16S rRNA gene phylogenies suggest that the novel isolates are different from the genera *Snodgrassella* and *Stenoxybacter*. Additionally, the ANI, G+C content, main fatty acids and phospholipid profile data supported the distinctiveness of the novel strain from genus *Snodgrassella*. Therefore, based on the data presented, the strains constitute a novel species of a novel genus within the family *Neisseriaceae*, for which the name *Populibacter corticis* gen. nov., sp. nov. is proposed. The type strain is 15-3-5^T^ (= CFCC 13594^T^ = KCTC 42251^T^).

## Introduction

In 2006, poplar bark canker with abundant white sour exudates was observed for the first time in China’s Henan and Shandong provinces. Initially diseased plants had stem or branch bark that cracked and exuded a frothy fluid, when the disease progressed, many cankers (50 to 150 by 3 to 8 cm, length by width) rapidly appeared. The bacterium *Lonsdalea quercina* subsp. *populi* was demonstrated to be the causal agent of these cankers [1]. *P*. × *euramericana* were found to develop bark cancer disease in China. Of four susceptible cultivars, *P*. × *euramericana* ‘Zhonglin 46’ was the most susceptible cultivar, followed bycultivars ‘74/76’ and *P*. *deltoids* ‘Zhonghe 1’[1]. A specific canker symptom has been detected in poplar (*P*.× *euramericana*) stands in the central part of Hungary since 2007 which was also observed in China. The bark of symptomatic trees is vertically cracked, and a sticky, brown-colored fluid oozes from the canker. Typically, huge numbers of the free-living, bacteriophagous nematode *Panagrellus redivivus* feed on the oozing fluid and on the creamy mass under the cracked bark. *Lonsdalea quercina* subsp. *populi* were isolated and identified in 2013 [2].

During our investigations on the bacterial diversity of the bark tissue of *P*. *× euramericana* canker, two isolates, 15-3-5^T^ and TQ2-3 belonging to the family *Neisseriaceae*, were isolated from symptomatic bark tissue of a *P*. *× euramericana* canker sample collected from Neihuang, Anyang City, and Taiqian, Puyang city, Henan Province, China in August 2010 and September 2015, respectively. To determine the taxonomic and phylogenetic position of this organism, the morphological, physiological, biochemical and chemotaxonomic characteristics, 16S rRNA gene sequences of the two novel isolates were analyzed. The results indicated that the novel isolates should be placed as a novel species of a novel genus in the family *Neisseriaceae*. Up to now, there are 15 valid published genera in family *Neisseriaceae* listed on www.bacterio.net. Some species of genus *Neisseria* are very important pathogen of human and animals, such as *Neisseria gonorrhoeae*, which is responsible for the sexually transmitted infection gonorrhea [3].

## Materials and methods

### Morphological, physiological and biochemical characterization

Observations of the colony morphology, motility and growth conditions were performed as previously described by Li *et al*. [4]. Catalase and oxidase activities were measured as recommended by Smibert and Krieg [5]. Gram staining was performed as reported by Schroeter [6]. Anaerobic growth was examined at 30°C for 1 week in anaerobic jars [7]. Hydrolysis of gelatin was tested according to the methods described by Smibert & Krieg [5]. Carbon source assimilation of two novel isolates and reference strain were tested by API 20NE (bioMérieux). API ZYM and API 50CHB/E (bioMérieux) were used for testing enzyme activities and acid production on two novel isolates. The assays were conducted according to the manufacturer’s instructions.

For the cellular fatty acid analysis, two novel strains were grown on tryptic soy agar (TSA, Difco) at 30°C and harvested in the exponential phase (for 24 h). Strain *Snodgrassella alvi* wkB2^T^ was grown on TSA at 30°C for 24 h under 3–5% CO_2_. Fatty acid methyl esters were extracted and analyzed according to the standard protocol of the Sherlock Microbial Identification System (MIDI, version 6.0) [8]. Chemotaxonomic analyses of polar lipids of strain 15-3-5^T^ were performed using 100 mg of freeze-dried cell material for two-dimensional thin-layer chromatography (TLC) as described by Minnikin *et al*. [9]. Menaquinones of strain 15-3-5^T^ were extracted according to the method of Collins *et al*. [10], analyzed by high performance liquid chromatography [11, 12] and confirmed by liquid chromatography/mass spectrometry (MS).

### 16S rRNA and acetate kinase gene sequence analysis

The 16S rRNA gene was amplified by PCR using primers 8F/1525R as described previously [13, 14]. After multiple sequence alignment using Clustal W, phylogenetic analysis with related reference species based on the maximum-likelihood and neighbor-joining methods was performed using MEGA5.1 by applying the Jukes-Cantor model including the proportion of invariable sites and gamma distribution (partial deletion of gaps/missing data treatment and a site coverage cut-off of 90%). The resulting trees were evaluated by 1000 bootstrap replicates [15, 16, 17]. For confirming phylogenetic relationships for the novel isolate (15-3-5^T^) and reference species of the family *Neisseriaceae*, the acetate kinase gene (ack) was also use to construct a maximum-likelihood tree using the amino acid sequence. The phylogenetic analysis was done as description above.

### Genome sequence analysis

The novel isolate (15-3-5^T^) was selected for genome sequencing as described by Romero-Hernández [18]. Pyrosequencing was performed using Ion Torrent technology with an Ion 316 v2 chip. The preliminary assembly was obtained using the MIRA software. Protein-coding genes were annotated by performing a BLAST search against the COGs database (http://www.ncbi.nlm.nih.gov/COG/).

Comparative sequence analysis of the genome core gene sequences was then used to discern phylogenetic relationships for the novel species (15-3-5^T^) and reference species of the family *Neisseriaceae*. The genome core gene sequences were identified and selected by the PanGP [19] software package, and a BLAST Matrix was constructed using a cutoff of 1e-5, 40% identity and 50% coverage. Aligned core genes were connected together with the same direction (MLST-like). The tree was based on alignment of the genome core gene sequences and was constructed using the neighbor-joining method (MEGA version 6.06) [20]. Nodal supports were assessed by 1000 bootstrap replicates.

### Average Nucleotide Identity (ANI)

ANI is a similarity measure between two genome sequences that may be used to replace DNA-DNA hybridization [21, 22]. In this study, the ANI among strain 15-3-5^T^, *Snodgrassella alvi* wkB2^T^ and *Stenoxybacter acetivorans* DSM19021^T^ was determined using the OrthoANI [23].

### Percentage of Conserved Proteins (POCP)

The percentage of conserved proteins (POCP) analysis was performed as described by Qin *et al*. [24]. In brief, the conserved proteins between a pair of genomes were determined by aligning all of the genome protein sequences of strain 15-3-5^T^ with all of the protein sequences of reference genome using the BLASTP program. The percentage of conserved proteins between two genomes was calculated as [(*C*1+*C*2)/(*T*1+*T*2)] · 100%, where *C*1 and *C*2 represent the conserved numbers of proteins in the two genomes being compared and *T*1 and *T*2 represent the total numbers of proteins in the two genomes being compared.

## Results

### Morphological, physiological and biochemical characterization

Colonies are milk white, opaque, circular, concave with entire margins, glossy and approximately 1–2 mm in diameter after 72 h growth at 30°C on TSA (pH 7). Growth occurs between 10 and 37°C and pH 5 to 10, with optimal growth at 28–30°C and pH 6.0–8.0. Growth is present under 0–3% (w/v) salinity. The detailed results were showed in species description and Table 1.

**Table 1:** Differential characteristics of *Populibacter corticis* gen. nov. sp. nov and reference species *Snodgrassella alvi* wkB2^T^.

Characteristic	*Populibacter corticis* gen. nov. sp. nov	*Snodgrassella alvi* wkB2^T^
Growth at 42°C	-	+
Growth in 5% CO_2_	-	+
Growth in air	+	-
DNA G+C content (mol%)	47.8	41.3*
Utilization of (API 20NE):		
Capric acid	+	-
Trisodium citrate	-	+
Activity of (API 20NE):		
Arginine dihydrolase	+	v
Urase	+	v
Major fatty acids (>10%)		
C_18:1_*ω*7*c*	24.0	43.5
C_16:1_*ω*7*c*/C_16:1_*ω*6*c*	27.2	4.1
Polar lipids	PE, PL, PG and L1-3	PE, PG, PNGL1 and PL1

Growth tests were performed on the TSA plate. All strains are positive for nitrate reductase, catalase-positive, and negative for the activities of oxidase, *β*-galactosidase, gelatinase and *β*-glucosidase and the production of indole. Malic acid is utilized by all strains, and it is negative for assimilation of d-glucose, l-arabinose, d-mannose, d-mannitol, *N*-acetyl-glucosamine, d-maltose, potassium gluconate, adipic acid, phenylacetic acid for all strains. PE = phosphatidylethanolamine, PG = phosphatidylglycerol, PNGL1 = phosphoaminoglycolipid, PL1 = phospholipid; L1-3, unknown lipids. V, variable reaction.

The major fatty acids detected in two isolates were C_16:0_, C_18:1_ *ω*7*c*, C_16:1_ *ω*7*c*/C_16:1_ *ω*6*c* (detailed in S1 Table), and were similar to the ones in the closely related genus *Snodgrassella*. However, the novel isolate can be distinguished from the related reference strain of genus *Snodgrassella* by the percentages of the main fatty acids (C_18:1_ *ω*7*c*, C_16:1_ *ω*7*c*/C_16:1_ *ω*6*c*, Table 1).

The major polar lipids of strain 15-3-5^T^ included phosphatidylethanolamine (PE), phospholipid (PL), phosphatidylglycerol (PG) and three unknown lipids (L1-3) (S1 Fig). The major lipids were the same as *Snodgrassella alvi* (Table 1) [25]. However, the absence of PNGL1, PL1 and presence of PL, L1-3 for the novel strain are useful to distinguish from *Snodgrassella alvi*. The quinone of the novel isolate was Q-8.

### 16S rRNA and ack gene sequence analysis

**Fig 1:**
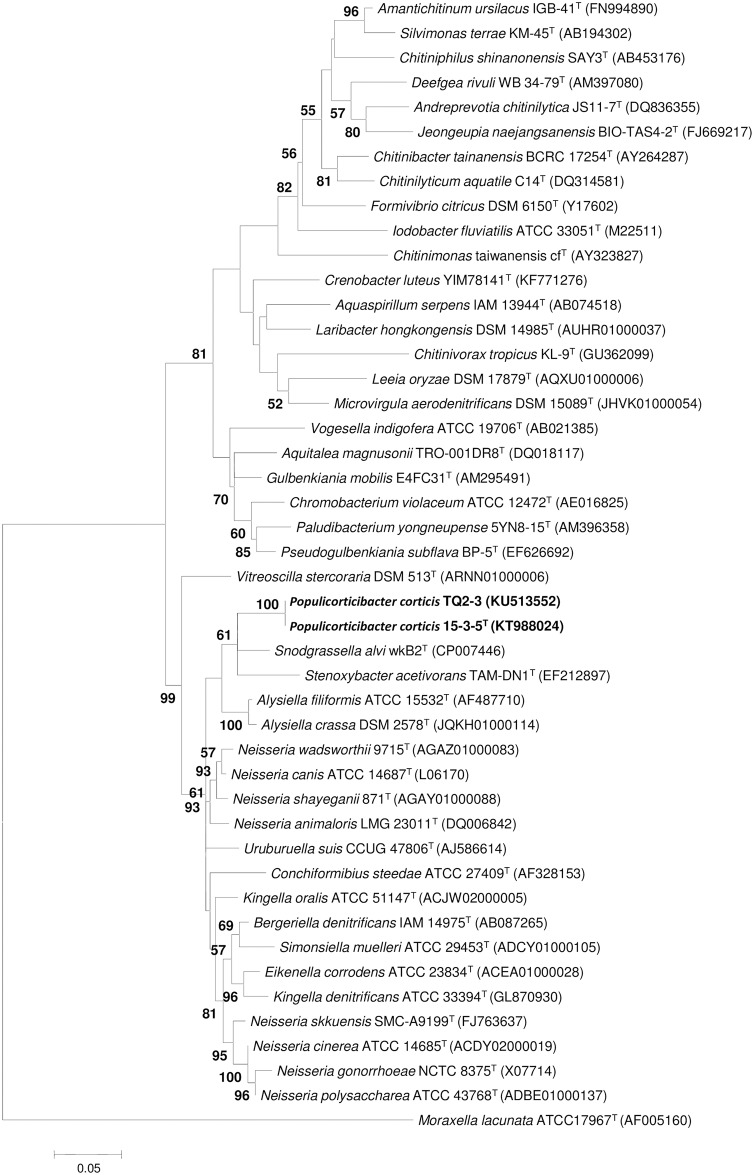
Maximum-likelihood tree showing phylogenetic relationships among members of the family *Neisseriaceae* and the novel isolates based on 16S rRNA gene sequences. *Moraxella lacunata* ATCC 17967^T^ was used as an outgroup. Only bootstrap values over 50% are shown. The scale bar corresponds to 0.05 substitutions per nucleotide site.

A 1421-bp sequence, corresponding to nucleotide positions 4–1457, was obtained. The GenBank accession numbers for the 16S rRNA gene sequence of strains 15-3-5^T^ and TQ2-3 are KT988024 and KU513552, respectively. Based on a comparison of 16S rRNA gene sequences, two novel isolates have identical sequences, and shared the highest sequence similarity with *Snodgrassella alvi* wkB2^T^ (94.5%), followed by *Neisseria animaloris* LMG 23011^T^ (94.3%), *Alysiella crassa* IAM 14969^T^ (94.2%), *Uruburuella testudinis* 07_OD624^T^(94.1%), and *Alysiella filiformis* ATCC 15532^T^ (94.0%), *Stenoxybacter acetivorans* TAM-DN1^T^ (94.0%), and less than 94% sequence similarity with all of the other species with validly published names (http://eztaxon-e.ezbiocloud.net) [26]. In the maximum-likelihood phylogenetic tree, two novel isolates clustered together with *Stenoxybacter acetivorans* TAM-DN1^T^, *Snodgrassella alvi* wkB2^T^ and *Alysiella filiformis* ATCC 15532^T^, forming one large branch, but the novel isolates branch was not related closely to any genus within the family *Neisseriaceae* (Fig 1). In the maximum-likelihood phylogenetic tree based on *ack* gene, the novel isolates were clustered together with *Snodgrassella alvi* wkB2^T^ and *Stenoxybacter acetivorans* TAM-DN6b and TAM-DN6b, and the novel isolates branch was close to *Snodgrassella alvi* wkB2^T^ branch (S2 Fig).

### Genome sequence analysis, ANI and POCP

The genome is 2361536 bp long, and the final assembly identified 131 large contigs. The genome accession number of strain 15-3-5^T^ is LQXR00000000. The max sequence length is 355320 bp. There are 2399 predicted genes, including 2013 protein-coding genes, 50 RNAs and 2 CRISPR repeats. The G+C content is 47.8%. The G+C content of the novel strain is higher than in *Snodgrassella alvi* wkB2^T^ (41.3%).

**Fig 2:**
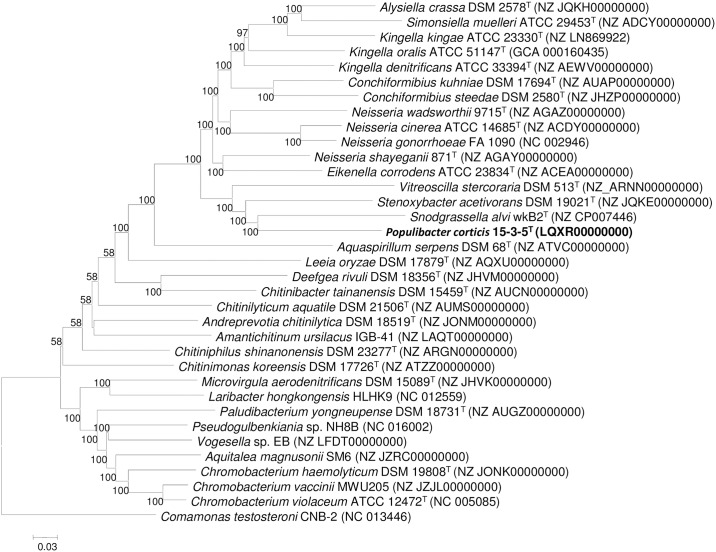
Phylogenetic tree showing the relationships among members of the family *Neisseriaceae* and the novel isolate. The tree is based on the alignment of genome core gene sequences and was constructed using the Neighbor-joining method (MEGA version 6.06). Nodal supports were assessed by 1000 bootstrap replicates. The scale bar is 0.03.

In the neighbor-joining phylogenetic tree based on the genome core gene sequence, 15-3-5^T^ was clustered together with *Snodgrassella alvi* wkB2^T^, *Stenoxybacter acetivorans* DSM 19021^T^ and *Vitreoscilla stercoraria* DSM 513^T^, forming one large branch (Fig 2). The ANI values between the 15-3-5^T^ and *Snodgrassella alvi* wkB2^T^ and *Stenoxybacter acetivorans* DSM19021^T^ are 70.1% and 71.8%, respectively (S2 Table). The value is lower than the proposed species boundary cut-off for ANI (95~96%) [23].

In POCP analysis, the novel isolate showed 59.8% and 56.2% POCP value with *Snodgrassella alvi* wkB2^T^ and *Stenoxybacter acetivorans* DSM 19021^T^. Furthermore, *Snodgrassella alvi* wkB2^T^ showed 53.9% POCP value with *Stenoxybacter acetivorans* DSM 19021^T^.

## Discussion

Two novel strains were isolated from poplar tree canker samples which are very complicated and special environment. Only two strains were isolated from more than 100 symptomatic canker bark samples because it is not the preponderant species in the poplar canker environment. Other researches showed that a sequence identity of 94.5% or lower for two 16S rRNA genes is strong evidence for distinct genera [27, 28]. In present study, the novel genus shared the highest sequence similarity with *Snodgrassella alvi* wkB2^T^ (94.5%). The 16S rRNA gene data suggest that the novel organism should represent a separate genus. The phylogenetic tree based on ack gene also indicated that the novel isolates were obviously distinguished from genera *Snodgrassella* and *Stenoxybacter* (S2 Fig). While, recent research by Qin et al. reported that generally, a percentage of conserved proteins (POCP) value of 50% can be proposed as a genus boundary for prokaryotic lineages being analyzed. While the interspecies POCP value ranged from 43% to 97%, and the average interspecies POCP values were 63% [24]. In their research, the interspecies and intergenera POCP analyses were performed only for 12 families/orders, including 97 genera and 235 species. With more and more bacterial genome sequencing, POCP value of 50% as a genus boundary for prokaryotic lineages should be further proved by using more genome data. In this study, the novel isolate showed the POCP identity values with *Snodgrassella alvi* wkB2^T^ and *Stenoxybacter acetivorans* DSM 19021^T^ are all more than 50% and less than 60% which are located in the boundary of the genera.

In the case of a strain or set of strains shown to be novel taxa, they should be characterized as comprehensively as possible [29]. In present study, the G + C content, major fatty acids and phospholipid profile data also support the distinctiveness of the novel species from genus *Snodgrassella*. Additionally, the novel species and *Snodgrassella alvi* can be differentiated from each other by the ability of the strain to utilize capric acid and citrate as unique carbon source to grow aerobicaly and anaerobically. (Table 1). Furthermore, the two novel strains can be differentiated from one another by DNA fingerprinting (ERIC, S3 Fig) and the activities of esterase lipase (C8), valine arylamidase, cystine arylamidase and trypsin. Therefore, two isolates are considered to represent a novel species representing a new genus of the family *Neisseriaceae*, for which the name *Populibacter corticis* gen. nov., sp. nov. is proposed

### Description of *Populibacter* gen. nov.

*Populibacter* gen. nov. (Po.pu.li.bac'ter L. n. *populus* a plant genus (poplar); N. L. masc. n. *bacter*, rod; N. L. masc. n. *Populibacter*, a rod from a poplar tree)

Cells are Gram-stain negative, aerobic, non-motile, rod-shaped. Cells are catalase-positive, oxidase-negative. Major fatty acids are C_16:0_, C_16:1_ *ω*7*c*/C_16:1_ *ω*6*c* and C_18:1_ *ω*7*c*. The main polar lipids are phosphatidylethanolamine (PE), phospholipid (PL), phosphatidylglycerol (PG) and three unknown lipids (L1-3). The Ubiquinone Q-8 is the only quinone type. The G+C content of the DNA of the type strain of the type species is 47.8mol%. Based on 16S rRNA sequence analysis, *Populibacter* belongs to the *Betaproteobacteria*. The type species is *Populibacter corticis*.

### Description of *Populibacter corticis* sp. nov.

*Populibacter corticis* sp. nov. (cor'ti.cis. L. gen. n. *corticis*, of the bark, referring to the poplar bark from which the type strain was isolated)

The description is as given for the genus with the following additions. Positive for the activities of nitrate reductase, arginine dihydrolase and urease, negative for the activities of *β*-galactosidase, gelatinase and *β*-glucosidase and the production of indole. Capric acid and malic acid are utilized as sole sources of carbon, and d-glucose, l-arabinose, d-mannose, d-mannitol, *N*-acetyl-glucosamine, d-maltose, potassium gluconate, adipic acid, citrate, phenylacetic acid are not utilized (API 20NE). It is negative for acid production from glycerol, erythritol, d-arabinose, l-arabinose, d-ribose, d-xylose, l-xylose, d-adonitol, methyl-*β* d-xylopyranoside, d-galactose, d-glucose, d-fructose, d-mannose, l-sorbose, l-rhamnose, dulcitol, inositol, d-mannitol, d-sorbitol, methyl-α d-mannopyranoside, methyl-α d-glucopyranoside, *N*-acetylglucosamine, amygdalin, arbutin, esculin ferric citrate, salicin, d-cellobiose, d-maltose, d-lactose, d-melibiose, d-saccharose, d-trehalose, inulin, d-melezitose, d-raffinose, amidon, glycogen, xylitol, gentiobiose, d-turanose, d-lyxose, d-tagatose, d-fucose, l-fucose, d-arabitol, l-arabitol, potassium gluconate, potassium 2-ketogluconate, potassium 5-ketogluconate (API 50CH system, bioMérieux). Positive for the activities (API ZYM) of alkaline phosphatase, esterase (C4), leucine arylamidase, acid phosphatase, and naphthol-AS-BI-phosphohydrolase. The strains are negative for the activities of lipase (C14), *α*-chymotrypsin, *α*-galactosidase, *β*-galactosidase, *β*-glucuronidase, α-glucosidase, *β*-glucosidase, *N*-acetyl-*β*-glucosaminidase, *α*-mannosidase, *α*-fucosidase. The activities of esterase lipase (C8), valine arylamidase, cystine arylamidase, trypsin are variable (the type strain is negative except for activities esterase lipase).

The type strain is 15-3-5^T^ (= CFCC 13594^T^ = KCTC 42251^T^) isolated from symptomatic bark tissue of *Populus × euramericana* canker.

## Supporting information

S1 Fig Polar lipid profiles of *Populibacter corticis* gen. nov. sp. nov. separated by two-dimensional TLC, detected by spraying with molybdatophosphoric acid reagent. PE, phosphatidylethanolamine; PL, phospholipid; PG, phosphatidylglycerol; PL, unknown lipids (1–3).
(JPG)

S2 Fig Maximum-likelihood tree showing phylogenetic relationships among members of the family *Neisseriaceae* and the novel isolate based on ack gene sequences. *Escherichia coli* K12 was used as an outgroup. Only bootstrap values over 50% are shown. The scale bar corresponds to 0.05 substitutions per nucleotide site.
(TIFF)

S3 Fig ERIC PCR fingerprint patterns of four novel strains. Lanes: 1, 15-3-5^T^, 2, TQ2-3, 3, 2 kb ladder.
(TIF)

S1 Table Comparison of fatty acid profiles of strain 15-3-5 ^T^ and *Snodgrassella alvi* wkB2^T^. (DOCX)

S2 Table Genome characteristics of the novel strain and two reference species. (DOCX)

## References

[1] Li Y, He W, Ren FJ, Guo LM, Chang J P, Cleenwerck I, et al A canker disease of *Populus* × *euramericana* in China, caused by *Lonsdalea quercina* subsp. *populi*. Plant Dis. 2014; 98(3): 368–378. 10.1094/PDIS-01-13-0115-RE 30708441

[2] Tóth T, Lakatos T, Koltay A. *Lonsdalea quercina* subsp. *populi* subsp. nov., isolated from bark canker of poplar trees. Int J Syst Evol Microbiol. 2013;63(Pt 6):2309–2313. 10.1099/ijs.0.042911-0 23159756

[3] Ryan KJ; Ray CG, eds. Sherris Medical Microbiology (4th ed*)* McGraw Hill 2004.

[4] Li Y, He W, Wang T, Piao CG, Guo LM, Chang JP, et al *Acinetobacter qingfengensis* sp. nov., isolated from canker bark of *Populus*× *euramericana*. Int J Syst Evol Microbiol. 2014; 64(Pt 3): 1043–1050. 10.1099/ijs.0.051995-0 24363297

[5] Smibert RM & Krieg NR. Phenotypic characterization In *Manual of Methods for General and Microbiology*, pp.607–654. Edited by Gerhardt P. Murray R.G.E. Wood W.A. & Krieg M N.R.. Washington, DC: American Society for Microbiology; 1994.

[6] Schroeter J. In *Kryptogamenflora von Schlesien*, Bd.3, Heft 3, *Pilze*. Edited by Cohn F.. Breslau: J.U. Kern’s Verlag; 1886.

[7] Gerhardt P, Murray RGE, Costilow RN, Nester EW, Wood WA, Krieg N R, Phillips GR. Manual of Methods for General Bacteriology. Washington, DC: American Society for Microbiology; 1981.

[8] Sasser M. Identification of bacteria by gas chromatography of cellular fatty acids, MIDI, Technical notes 101. Newark, DE: MIDI Inc; 1990.

[9] Minnikin DE, O’Donnell AG, Goodfellow M, Alderson G, Athalye M, Schaal A, Parlett JH. An integrated procedure for the extraction of isoprenoid quinones and polar lipids. J Microbiol Meth. 1984; 2(5): 233–241.

[10] Collins MD, Pirouz T, Goodfellow M, Minnikin DE. Distribution of menaquinones in *actinomycetes* and *corynebacteria*. J Gen Microbiol. 1977; 100(2): 221–230. 10.1099/00221287-100-2-221 894261

[11] Du HJ, Zhang YQ, Liu HY, Su J, Wei YZ, Ma BP, et al *Allonocardiopsis opalescens* gen. nov., sp. nov., a new member of the suborder *Streptosporangineae*, from the surface-sterilized fruit of a medicinal plant. Int J Syst Evol Microbiol. 2013; 63(Pt 3): 900–904. 10.1099/ijs.0.041491-0 22634703

[12] Groth I, Schumann P, Rainey FA, Martin K, Schuetze B, Augsten K. Demetria terragena gen. nov., sp. nov., a new genus of actinomycetes isolated from compost soil. Int J Syst Bacteriol. 1997; 47(4):1129–1133. 10.1099/00207713-47-4-1129 9336919

[13] Baker GC, Smith JJ, Cowan DA. Review and reanalysis of domainspecific 16S primers. J Microbiol Meth. 2003; 55(3): 541–555. 10.1016/j.mimet.2003.08.009 14607398

[14] Lane DJ. 16S/23S rRNA sequencing In *Nucleic Acid Techniques in Bacterial Systematics*, pp. 115–175. Edited by Stackebrandt E. and Goodfellow M.. Chichester: Wiley; 1991.

[15] Felsenstein J. Confidence limits on phylogenies: an approach using the bootstrap. Evolution. 1985; 39(4): 783–791. 28561359 10.1111/j.1558-5646.1985.tb00420.x

[16] Tamura K, Peterson D, Peterson N, Stecher G, Nei M, Kumar S. MEGA5: Molecular evolutionary genetics analysis using maximum likelihood, evolutionary distance, and maximum parsimony methods. Mol Biol Evol. 2011; 28(10): 2731–2739. 10.1093/molbev/msr121 21546353 PMC3203626

[17] Thompson JD, Gibson TJ, Plewniak F, Jeanmougin F, Higgins D G. The Clustal X windows interface: flexible strategies for multiple sequence alignment aided by quality analysis tools. Nucleic Acids Res. 1997; 25(24): 4876–4882. 9396791 10.1093/nar/25.24.4876 PMC147148

[18] Romero-Hernández B, Tedim AP, Sánchez-Herrero JF, Librado P, Rozas J., Muñoz G. et al *Streptococcus gallolyticus* subsp. *gallolyticus* from human and animal origins: genetic diversity, antimicrobial susceptibility, and characterization of a vancomycin-resistant calf isolate carrying a vanA-Tn1546-like element. Antimicrobial agents and chemotherapy. 2015; 59(4): 2006–2015. 10.1128/AAC.04083-14 25605355 PMC4356806

[19] Zhao Y, Jia X, Yang J, Ling Y, Zhang Z, Yu J, et al PanGP: a tool for quickly analyzing bacterial pan-genome profile. Bioinformatics, 2014; 30(9):1297–1299. 10.1093/bioinformatics/btu017 24420766 PMC3998138

[20] Tamura K, Stecher G, Peterson D, Filipski A, Kumar S. MEGA6: molecular evolutionary genetics analysis version 6.0. Mol Biol Evol. 2013; 30(4): 2725–2729 24132122 10.1093/molbev/mst197 PMC3840312

[21] Goris J, Konstantinidis KT, Klappenbach JA, Coenye T, Vandamme P, Tiedje JM. DNA-DNA hybridization values and their relationship to whole-genome sequence similarities. Int J Syst Evol Microbiol. 2007; 57(1): 81–91. 17220447 10.1099/ijs.0.64483-0

[22] Richter M, Rosselló-Móra R. Shifting the genomic gold standard for the prokaryotic species definition. PNAS. 2009; 106(46): 19126–19131. 19855009 10.1073/pnas.0906412106 PMC2776425

[23] Lee I, Kim YO, Park SC, Chun J. OrthoANI: An improved algorithm and software for calculating average nucleotide identity. Int J Syst Evol Microbiol. 2016; 66: 1100–1103. 26585518 10.1099/ijsem.0.000760

[24] Qin QL. Xie BB, Zhang XY, Chen XL, Zhou BC, Zhou JZ, et al A proposed genus boundary for the prokaryotes based on genomic insights. *Journal of Bacteriology*; 196: 2210–2215. 10.1128/JB.01688-14 24706738 PMC4054180

[25] Kwong W K, Moran NA. Cultivation and characterization of the gut symbionts of honey bees and bumble bees: description of *Snodgrassella alvi* gen. nov., sp. nov., a member of the family *Neisseriaceae* of the *Betaproteobacteria*, and *Gilliamella apicola* gen. nov., sp. nov., a member of Orbaceae fam. nov., Orbales ord. nov., a sister taxon to the order ‘*Enterobacteriales*’ of the Gammaproteobacteria. Int J Syst Evol Microbiol. 2013; 63(Pt 6): 2008–2018. 10.1099/ijs.0.044875-0 23041637

[26] Kim OS, Cho YJ, Lee K, Yoon SH, Kim M, Na H, et al Introducing EzTaxon-e: a prokaryotic 16S rRNA gene sequence database with phylotypes that represent uncultured species. Int J Syst Evol Microbiol. 2012; 62(Pt 3): 716–721. 10.1099/ijs.0.038075-0 22140171

[27] Yarza P, Richter M, Peplies J, Euzeby J, Amann R, Schleifer KH, et al The All-Species Living Tree project: A 16S rRNA-based phylogenetic tree of all sequenced type strains. Syst Appl Microbiol. 2008; 31: 241–250. 10.1016/j.syapm.2008.07.001 18692976

[28] Yarza P, Yilmaz P, Pruesse E, Glöckner FO, Ludwig W, et al Uniting the classification of cultured and uncultured bacteria and archaea using 16S rRNA gene sequences. Nat Rev Micro. 2014;12: 635–645. 10.1038/nrmicro3330 25118885

[29] Tindall BJ, Rosselló-Móra R, Busse HJ, Ludwig W, Kämpfer P. Notes on the characterization of prokaryote strains for taxonomic purposes. Int J Syst Evol Microbiol. 2010; 60: 249–266. 10.1099/ijs.0.016949-0 19700448

