# Supplementary figures and images for: Phylogenetic analysis of family Neisseriaceae based on genome sequences and description of Populibacter corticis gen. nov., sp. nov., a member of the family Neisseriaceae, isolated from symptomatic bark of Populus × euramericana canker
Source: PLoS One. 2017 Apr 13;12(4):e0174506. doi: 10.1371/journal.pone.0174506 (PMC5390963; doi:10.1371/journal.pone.0174506)

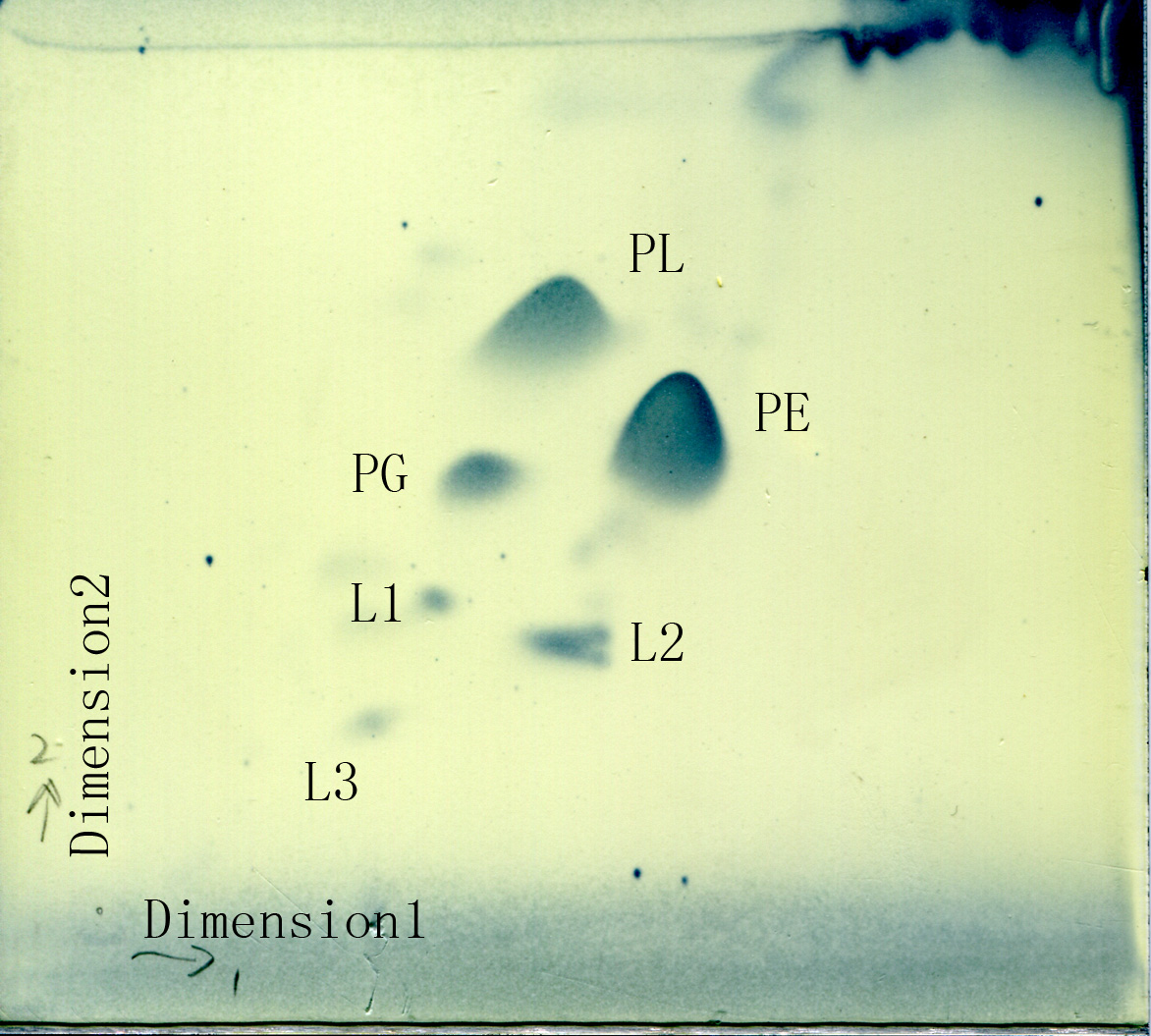

Supplement: S1 Fig — PE, phosphatidylethanolamine; PL, phospholipid; PG, phosphatidylglycerol; PL, unknown lipids (1–3). (JPG) [file pone.0174506.s001.jpg]

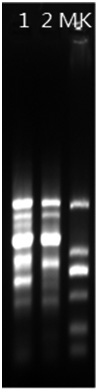

Supplement: S3 Fig — Lanes: 1, 15-3-5T, 2, TQ2-3, 3, 2 kb ladder. (TIF) [file pone.0174506.s003.tif]
