# Supplementary material for: Phylogenetic analysis of family Neisseriaceae based on genome sequences and description of Populibacter corticis gen. nov., sp. nov., a member of the family Neisseriaceae, isolated from symptomatic bark of Populus × euramericana canker
Source: PLoS One. 2017 Apr 13;12(4):e0174506. doi: 10.1371/journal.pone.0174506 (PMC5390963; doi:10.1371/journal.pone.0174506)
Supplement: S1 Table — (DOCX) [file pone.0174506.s004.docx]

S1 table. Comparison of fatty acid profiles of novel species and *Snodgrassella alvi* wkB2^T^

Strains: 1, 15-3-5 ^T^**;** 2, TQ3-2; *Snodgrassella alvi* wkB2^T^;

| Major fatty acids (%) | **1** | 2 | 3 |
| --- | --- | --- | --- |
| C_12:0_ | 6.9 | 6.7 | 7.9 |
| C_12:0_ 3OH | 5.1 | 5.6 | 6.2 |
| C_14:0_ | 5.9 | 6.1 | 6.4 |
| C_14:0_ 3OH/ C_16:1_ isoI | 2.1 | 3.5 | 0.6 |
| C_16:0_ | 28.3 | 26.1 | 29.6 |
| C_18:1_ *ω*7*c* | 23.7 | 24.2 | 43.5 |
| C_16:1_ *ω*7*c*/C_16:1_ *ω*6*c* | 27.5 | 26.9 | 4.1 |
