# Supplementary material for: Phylogenetic analysis of family Neisseriaceae based on genome sequences and description of Populibacter corticis gen. nov., sp. nov., a member of the family Neisseriaceae, isolated from symptomatic bark of Populus × euramericana canker
Source: PLoS One. 2017 Apr 13;12(4):e0174506. doi: 10.1371/journal.pone.0174506 (PMC5390963; doi:10.1371/journal.pone.0174506)
Supplement: S2 Table — (DOCX) [file pone.0174506.s005.docx]

**S2 Table.** Genome characteristics of the novel strain and two reference species

1, 15-3-5 ^T^**;** 2, *Snodgrassella alvi* wkB2^T^; 3, *Stenoxybacter acetivorans* DSM 19021^T^

|  | **1** | 2 | 3 |
| --- | --- | --- | --- |
| Genome size (Mb) | 2.4 | 2.5 | 2.6 |
| DNA G+C content (%) | 47.8 | 41.3 | 45.6 |
| Total genes | 2399 | 2297 | 2490 |
| RNA genes | 50 | 72 | 45 |
| Protein-coding genes | 2013 | 2198 | 2263 |
| **ANI(%)** |  |  |  |
| 1 |  | 70.1 | 71.9 |
| 2 | 70.1 |  | 69.9 |
| 3 | 71.9 | 70.0 |  |
